# Recurrent Pneumonia? A Case of Availability Bias and Anchoring

**DOI:** 10.7759/cureus.11088

**Published:** 2020-10-22

**Authors:** Courtney M Rentas, Ryan Goetz, Takudzwa Mkorombindo, Sanjiv Bajaj, Robert Centor

**Affiliations:** 1 Department of Medicine, The University of Alabama School of Medicine, Birmingham, USA; 2 Department of Radiology, Birmingham Veterans Affairs Medical Center, Birmingham, USA

**Keywords:** invasive mucinous adenocarcinoma, recurrent pneumonia, availability bias, anchoring bias

## Abstract

Invasive mucinous adenocarcinoma is a multi-centric adenocarcinoma that accounts for less than 5% of all lung cancer diagnoses. The most common presenting symptoms (cough, sputum production, and chest pain) in conjunction with its radiographic findings (patchy, multi-lobar infiltrates) make invasive mucinous adenocarcinoma challenging to distinguish from both infectious and inflammatory pneumonia. However, due to its aggressive nature, invasive mucinous adenocarcinoma should be considered if a presumed case of pneumonia lacks symptoms of infection (e.g., fever, leukocytosis) and/or does not respond to antibiotics. We report the case of a 75-year-old male who was admitted in the setting of a presumed case of recurrent pneumonia, which had failed to respond to prior antibiotic therapy. Further workup, including trans-bronchial biopsy, confirmed mucinous adenocarcinoma with a lepidic pattern. This case highlights the importance of establishing a broad differential in the setting of unresolved pneumonia following appropriate antibiotic coverage.

## Introduction

Invasive mucinous adenocarcinoma is a multi-centric adenocarcinoma with a characteristic lepidic pattern [1]. Its often patchy, multi-lobar presentation makes it challenging to differentiate from multi-focal pneumonia radiographically [2]. Additionally, because patients commonly present with symptoms of cough with sputum production and chest pain, it becomes increasingly difficult to differentiate it from both infectious and inflammatory pneumonia (e.g., cryptogenic organizing pneumonia, eosinophilic pneumonia) [2,3]. Despite these apparent similarities, the course of the disease is significantly different from those of the previously mentioned etiologies and warrants prompt recognition and treatment by physicians, i.e., it should be considered in cases of unresolved pneumonia.

## Case presentation

**Figure 1:**
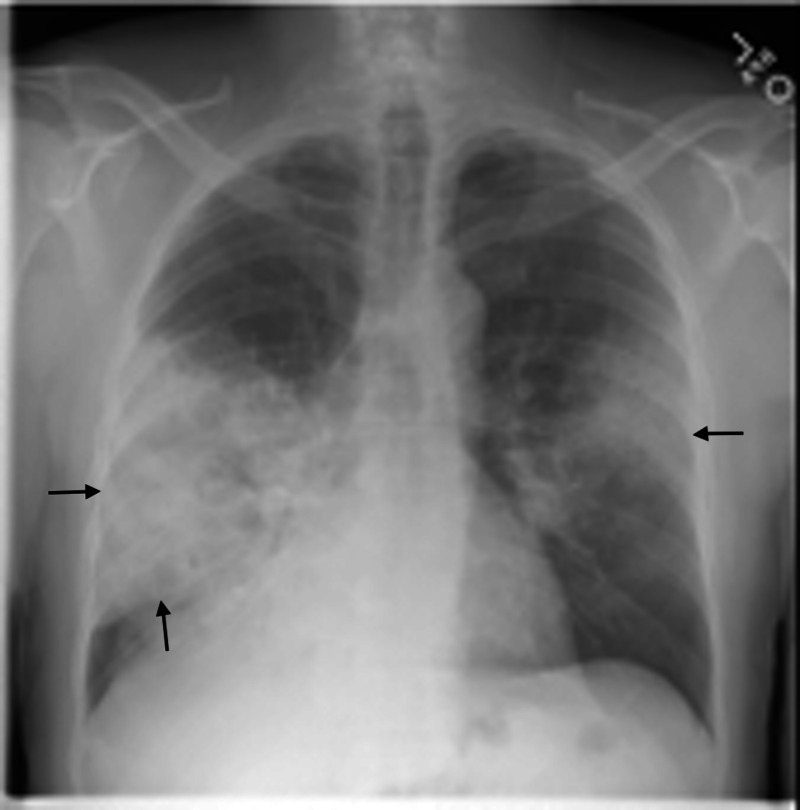
Chest X-ray four months prior to diagnosis Arrows indicate areas of focal consolidation involving the right middle lobe, portions of the right lower lobe, and left midlung

A 75-year-old man with a history of hypertension and allergic rhinitis had initially presented one year prior to admission to his primary care physician with a non-productive cough and had been treated empirically for post-nasal drip. There had been limited response to this treatment. Three months prior to the presentation, the patient had sought treatment for a worsening, now-productive cough with thick white sputum. At that time, the patient had denied fever, chills, dyspnea, and chest pain but acknowledged intermittent episodes of small-volume hemoptysis. The patient had a 20 pack-year history of smoking but had quit 40 years prior. Chest radiography had revealed multi-lobar consolidations (Figure 1). A complete blood count (CBC) had shown no leukocytosis. A presumptive diagnosis of bacterial pneumonia had been made, and the patient had been treated with levofloxacin and azithromycin.

**Figure 2:**
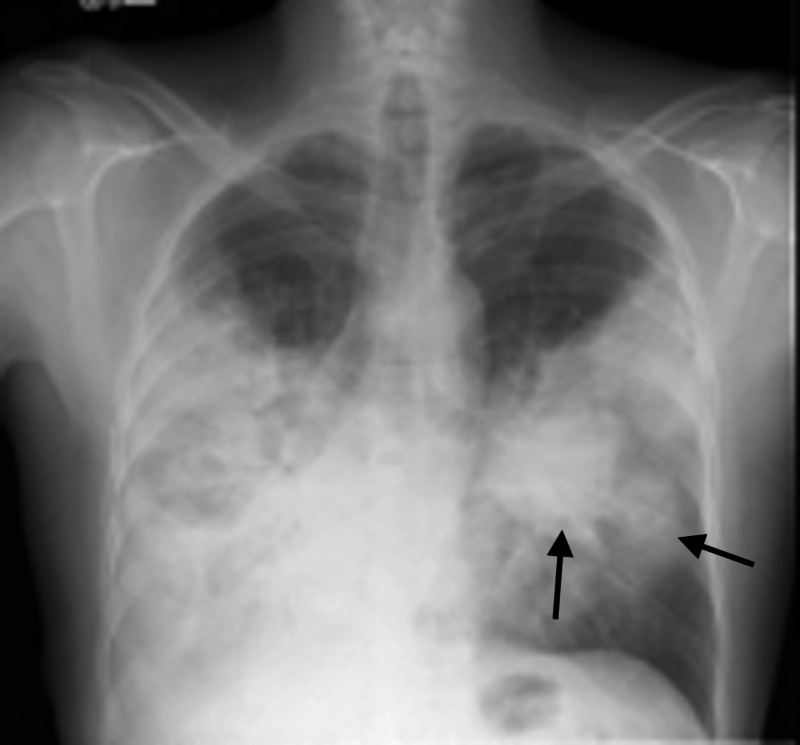
Chest X-ray three months prior to diagnosis Interval worsening of previously noted consolidations when compared to initial X-ray three weeks prior. Arrows identify nodules

**Figure 3:**
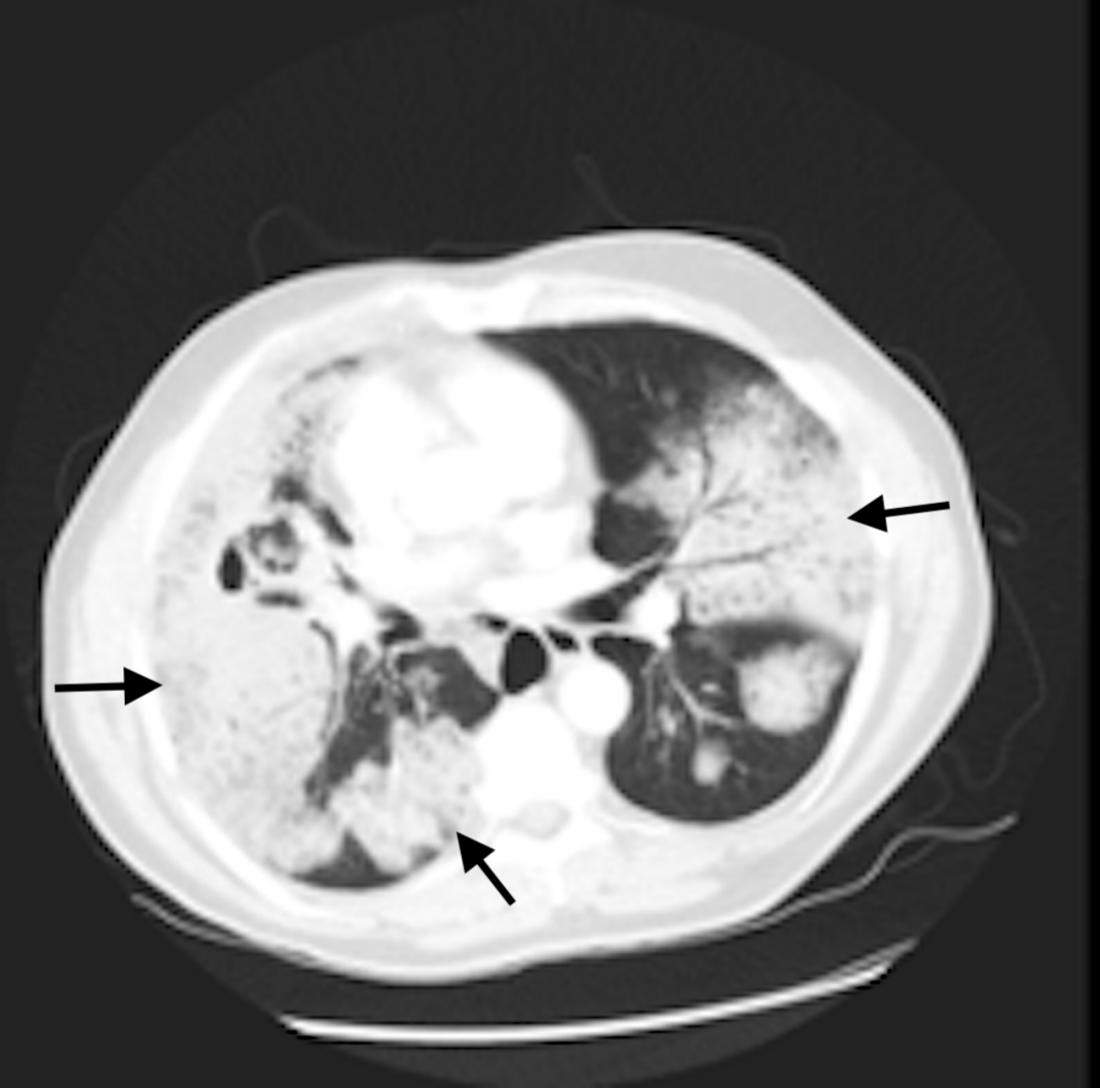
CT Scan 2.5 months prior to diagnosis CT imaging depicts multi-focal consolidation (arrows) with air bronchograms and some alveolar sparing CT: computed tomography

Three weeks later, despite the antibiotic regimen, the patient continued to have a persistent cough, now productive of thick yellow sputum. A repeat chest radiograph was obtained and revealed worsening consolidation with nodular areas concerning for worsening pneumonia vs. malignancy (Figure 2). A chest CT revealed multi-lobar consolidation with air bronchograms (Figure 3), prompting evaluation by the pulmonary service, and admission for IV antibiotics.

**Figure 4:**
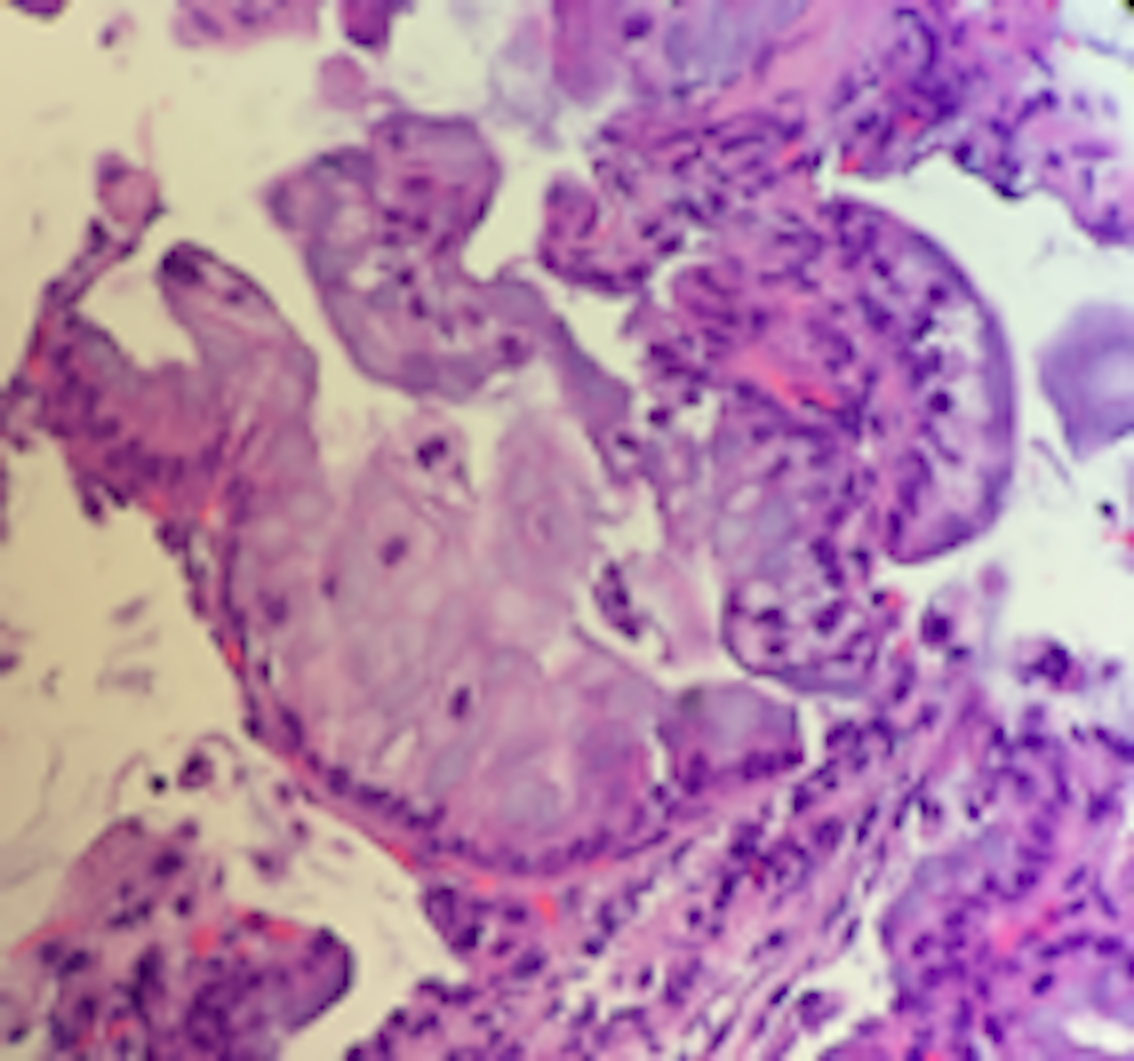
Transbronchial biopsy at diagnosis Transbronchial biopsy findings confirm the diagnosis of mucinous adenocarcinoma with lepidic pattern

On admission, additional history was obtained, revealing a 15-lb weight loss in the past three months. He denied night sweats, recent domestic or international travel, animal exposure, potential work-related exposures, arthralgias, muscle weakness, or sick contacts. On physical exam, rales were auscultated bilaterally, egophony was noted over the right lower and middle lung fields, and there was dullness to percussion over the right middle lung field. A bronchoscopy with bronchoalveolar lavage (BAL) and transbronchial biopsy were performed, and pathology was consistent with a diagnosis of mucinous adenocarcinoma with lepidic pattern (Figure 4).

## Discussion

Non-infectious etiologies, including neoplasm, inflammatory, and drug-induced etiologies, can present as non-resolving pneumonia, necessitating a thorough workup for patients whose radiographic findings persist following appropriate antibiotic therapy. Slow-resolving pneumonia is defined radiographically, with imaging showing less than 50% infiltrate clearance within two weeks of presentation or failure of complete clearance within four weeks of presentation following antibiotic therapy [4,5].

The workup for non-resolving pneumonia relies heavily on bronchoscopy with BAL with cultures for bacteria, legionella, fungi, and mycobacterium. If clinical findings do not support an infectious etiology (afebrile, lack of purulent sputum, no leukocytosis), further studies including imaging and, if indicated, tissue sampling (i.e., transbronchial lung biopsy) should be obtained [6].

Both availability and anchoring biases likely played a role in our patient’s delayed diagnosis. On his initial chest X-ray, bilateral infiltrates were seen, prompting the clinical diagnosis of pneumonia, even though our patient’s presentation pattern lacked many of the salient features of pneumonia. Furthermore, when the patient presented again with worsening pulmonary symptoms (still without signs of systemic inflammation to suggest pneumonia), the inclination was to consider this bacterial pneumonia refractory for outpatient antibiotic therapy and admit the patient for IV antibiotics. Heuristics and other forms of system 1 decision making play a role in many of our clinical decisions [7,8]. However, at times, these may lead us astray, and we must be mindful of instances when the presentation pattern and treatment outcomes do not match our knowledge of the disease. During these times, it is essential to take a step back and systematically consider the clinical presentation and differential diagnosis. Our patient’s clinical presentation and lack of response to treatment were not consistent with a clinical diagnosis of pneumonia. Additional imaging and tissue sampling were obtained, thereby cinching the diagnosis of mucinous adenocarcinoma.

## Conclusions

This case serves to highlight the importance of integrating clinical and radiographic findings to pursue an essential workup in patients with “recurrent pneumonia”.
